# NIN Is Involved in the Regulation of Arbuscular Mycorrhizal Symbiosis

**DOI:** 10.3389/fpls.2016.01704

**Published:** 2016-11-16

**Authors:** Bruno Guillotin, Jean-Malo Couzigou, Jean-Philippe Combier

**Affiliations:** Laboratoire de Recherche en Sciences Végétales, Université de Toulouse, Centre National de la Recherche Scientifique, Unités Propres de ServiceCastanet Tolosan, France

**Keywords:** *Rhizophagus irregularis*, *Medicago truncatula*, symbiosis, arbuscular mycorrhiza, infection, common symbiotic signaling pathway (CSSP), *Nodule Inception* (*NIN*)

## Abstract

Arbuscular mycorrhizal (AM) symbiosis is an intimate and ancient symbiosis found between most of terrestrial plants and fungi from the *Glomeromycota* family. Later during evolution, the establishment of the nodulation between legume plants and soil bacteria known as rhizobia, involved several genes of the signaling pathway previously implicated for AM symbiosis. For the past years, the identification of the genes belonging to this Common Symbiotic Signaling Pathway have been mostly done on nodulation. Among the different genes already well identified as required for nodulation, we focused our attention on the involvement of Nodule Inception (*NIN*) in AM symbiosis. We show here that *NIN* expression is induced during AM symbiosis, and that the *Medicago truncatula nin* mutant is less colonized than the wild-type *M. truncatula* strain. Moreover, *nin* mutant displays a defect in the ability to be infected by the fungus *Rhizophagus irregularis*. This work brings a new evidence of the common genes involved in overlapping signaling pathways of both nodulation and in AM symbiosis.

## Introduction

Throughout evolution, plants have adapted in order to cope with several stresses and among them, nutrient deprivation. At least 80% of the terrestrial plants have the ability to undergo a symbiosis with a soil monophyletic fungal lineage (*Glomeromycota*) called arbuscular mycorrhizal fungi (AMF) (Smith and Read, 2008). This mutually beneficial interaction is characterized by reciprocal exchanges in which the fungus provides soil mineral nutrients from its hyphal network to the plant in exchange for photosynthetically produced carbohydrates. This symbiosis with the AMF has an extremely long-term co-evolutionary history since its appearance approximately 450 million years ago, and it has been proposed to have played a key role in the colonization of terrestrial environment by plants (Redecker et al., 2000). The establishment of this symbiosis starts with an exchange of molecular signals between the two partners. The plant roots exude different types of molecules of the strigolactone family. These molecules play an important role by stimulating the hyphae branching and the metabolism of the fungus (Akiyama et al., 2005; Besserer et al., 2006, 2008). From the other side, AM fungi produce different types of molecules such as lipo-chitooligosaccharides (Myc-LCOs) and chitin oligosaccharides (COs). Myc-LCOs recognition by plant receptors activates the Common Symbiotic Signaling Pathway (CSSP; Kistner and Parniske, 2002; Oldroyd, 2013) and promote lateral root formation, more suitable for fungal colonization (Oláh et al., 2005; Gutjahr et al., 2009; Maillet et al., 2011; Genre et al., 2013). Following these early signaling events, the fungus develops to reach the plant root epidermis, creates specific entrance structures called hyphopodia and colonizes the root inter- and intra-cellularly. Finally, the fungus forms in cortical cells highly branched structures, called arbuscules, which are the real headquarter of the dual nutrient exchange between the plant and the fungus.

More recently during evolution, Leguminous plant lineage has developed the ability to realize an intracellular symbiosis with several bacteria collectively known as rhizobia (Werner et al., 2014). Rhizobia are hosted in root-derived structures called nodules. This symbiosis ultimately allows the reduction of atmospheric nitrogen into ammonium, used for plant nutrition. Establishment of this nitrogen-fixing symbiosis appeared to rely on the same gene set used for AM symbiosis, thus bringing the CSSP concept (also known as the SYM pathway) (Kistner and Parniske, 2002; Oldroyd, 2013).

It has been shown during nodulation that among the numerous transcription factors known to be involved in the CSSP, the flawing of at least one of them results in a complete incapacity for the plant to form nodules. Interestingly, mutants of some of these transcription factors, like *IPD3*, *NSP1*, or *NSP2*, keep their ability to form AM symbiosis, but with less efficiency (Horváth et al., 2011; Maillet et al., 2011; Delaux et al., 2013; Xue et al., 2015).

Thus, the precise phenotyping during the study of AM interaction remains difficult when it comes to very finely tuned regulations. In particular, highly concentrated fungal inocula are known to hide AM symbiosis reduced phenotypes (Delaux et al., 2013). For this reason, a lot of genes known to be crucial for nodulation, have not been characterized to be part of AM symbiosis to date. Therefore, it is only very recently that two GRAS transcription factors NSP1 and NSP2 (Nodulation Signaling Pathway), have been shown to be components of the MYC signaling pathway (Maillet et al., 2011; Lauressergues et al., 2012; Delaux et al., 2013). These transcriptional regulators act to coordinate the expression of nodulation-associated genes such as Early Nodulin 11 (ENOD11), a marker gene for Nod Factor-induced responses (Andriankaja et al., 2007). Several other transcriptional factors such as ERN1, ERN2, NF-YA1, NF-YA2 appear to play similar roles (Andriankaja et al., 2007; Middleton et al., 2007; Laloum et al., 2014). Among them NIN (Nodule Inception) has been extensively studied for its roles during early and late stages of nodulation, and especially its role as a repressor of ENOD11 (Schauser et al., 1999; Marsh et al., 2007; Soyano et al., 2013, 2014; Vernié et al., 2015). Indeed *NIN* is crucial for the induction of Nodulation Pectate Lyase (NPL), required for rhizobial infection of the epidermis (Xie et al., 2012), as well as for the induction of NF-YA1 and NF-YA2, associated with cortical cell divisions (Soyano et al., 2013). Later, *NIN* is also involved in the control of the nodule number via the induction of CLE peptides, key factors of the autoregulation process (Oka-Kira and Kawaguchi, 2006; Soyano and Hayashi, 2014).

Because NIN acts downstream of NSP1 and NSP2 during nodulation (Marsh et al., 2007), and because these latter genes have been shown to play a role during the control of fungal colonization (Maillet et al., 2011; Lauressergues et al., 2012; Delaux et al., 2013), we analyzed the role of NIN during the mycorrhization process. We show here that *NIN* expression is up-regulated by exogenous Myc-LCOs treatment as well as during mycorrhization. Moreover *NIN* loss of function mutant presented a reduced general mycorrhization but without affecting arbuscule morphogenesis.

## Materials and Methods

### Biological Material

*Medicago truncatula* Gaertn ‘Jemalong’ genotype A17 and *nin1-1* (Marsh et al., 2007) seed were used in this study. Seeds coats were first scarified by incubation in concentrated H_2_SO_4_ (10 min), then seeds were surface-sterilized using 9% NaClO (2 min) before to be extensively washed with deionized sterile water and germinated on agar plates in the dark for 5 days at 4°C. Plants were cultivated in 250 mL pots filled with Oil-Dri US-special substrate (Damolin^1^) for 5–9 weeks in a growth chamber (16/8 h day/night, 25°C/23°C, 30/80% hygrometry, 260–300 μmol m^-2^s^-1^) and watered every 2 days with modified Long Ashton medium containing a low concentration of phosphate (7.5 μM; Balzergue et al., 2011). For mycorrhization experiments, plants were inoculated with *Rhizophagus irregularis* DAOM 197198 sterile spores (400 spores per liter of substrate) purchased from Agronutrition (Carbone, France).

The quantification of the number of fungal infection points and the measures of the colonization length were done on the total root system of 2 weeks old plants growing in 50 ml Falcon tubes containing 50 spores of *R. irregularis* and cultivated in the similar conditions as above. The experiment was done on eight plants per conditions and repeated three times.

For *in vitro* culture, germinated seedlings were grown on solidified Fahraeus medium (Fahraeus, 1957), containing a low concentration of phosphate (7.5 μM) and a high concentration of nitrogen (10 mM NH_4_NO_3_), to avoid crosstalk with the NOD pathway, 8% agar (KALYS BIOTECH, AGAR HP 696) in 12 cm square plates (eight seedlings per plate) in a growth chamber.

### Bioactive Chemicals

Myc-LCOs were provided by Eric Samain, Sébastien Fort and Sylvain Cottaz (CERMAV, Grenoble, France), and used as an equimolar mix of the four (Syn)Myc-LCOs: LCO-IV(C16 : 0), LCO-IV(C16 : 0,S), LCO- IV(C18 : 1D9Z), and LCO-IV(C18 : 1D9Z,S), described in Maillet et al. (2011), at a final concentration of 10^-8^ M. COs with four residues (CO4; Genre et al., 2013) were used at a final concentration of 10^-8^ M in the same conditions as Myc-LCOs. The plants were treated for 12 h previous collecting the roots for qRT-PCR analyses.

### Quantitative Reverse Transcription Polymerase Chain Reaction (qRT-PCR) Analyses

Total RNA of *M. truncatula* roots were extracted using the RNeasy Plant Mini Kit (Qiagen). The reverse transcription (RT) was performed using the SuperScript II Reverse Transcriptase (Invitrogen) on 1 μg of total RNA, after removal of potential contaminating DNA, using RQ1 DNase (Promega). For the experiment comparing the mycorrhizal and non-mycorrhizal roots, six independent plants were analyzed per condition. For the experiments with molecule treatments, 10 independent plants were pooled per replicate. Three replicates (*n* = 3) were performed with two technical replicates each. Each experiment has been repeated two (myc- vs. myc+ experiment) to three times (others). A representative experiment of the two or three has been shown in figures. The quantitative polymerase chain reaction (qPCR) amplifications were conducted on a Roche LightCycler 480 System (Roche Diagnostics; Lauressergues et al., 2012). Ubiquitin and EF1 were used to normalize relative gene expression. Relative expression of genes of interest in the control roots was put at 100. Primers used described in **Supplementary Table S1**.

Nodule Inception gene expression was also analyzed using the Medicago gene atlas http://mtgea.noble.org/v3/, probe set: Mtr.28094.1.S1_at.

### Mycorrhizal Phenotyping and Fungus Staining

Roots were cleared in 10% (w/v) solution of KOH, 8 min at 95°C and rinsed in water. The roots were then stained with Schaeffer black ink as described in Vierheilig et al. (1998). The percentage of mycorrhization was established using the grid intersect method described by Giovannetti and Mosse (1980) and with two additional mycorrhization indices: F, mycorrhization frequency and a, arbuscule abundance in colonized root sections, according to Trouvelot et al. (1986). The quantification of the number of fungal infection points and the measures of the colonization length were done by selecting and including under lamella (in 30% water/glycerol solution) all mycorrhized regions of each plant. Images were then acquired at high resolution using a Nanozoomer 2.0 HT (Hamamatsu, Japan) and images were analyzed with the NDP view 2.5 software.

### Statistical Analyses

The mean values of relative gene expression or mycorrhization rates were compared by using the Kruskal-Wallis test and, when significant, a pairwise comparison was made using the non-parametric Mann–Whitney test. The error bars represent the standard error of the mean (SEM). The asterisks indicate significant differences (*P* < 0.05).

## Results

### Mycorrhization and Myc-LCOs Induce *NIN* Expression

**FIGURE 1:**
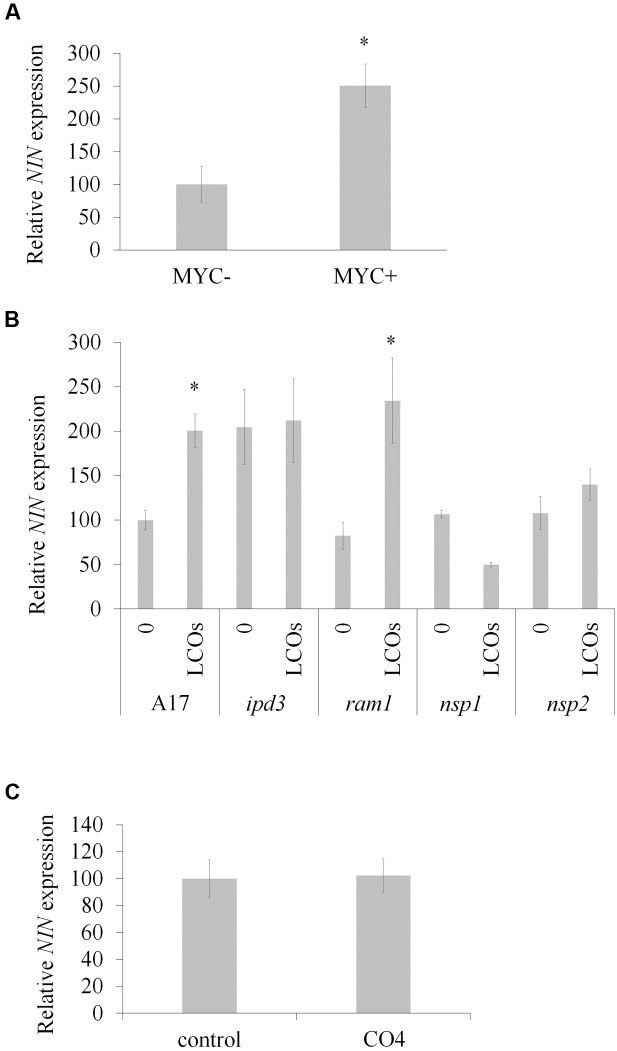
**Nodule Inception (*NIN)* expression during AM symbiosis.** **(A)** Quantification of *NIN* expression by qRT-PCR in non-inoculated (MYC-) and roots inoculated with *R. irregularis* (MYC+) and cultivated for 9 weeks (*n* = 6 independent plants). **(B)** Quantification of *NIN* expression by qRT-PCR in wild type (WT; A17) and mutant lines treated or not with a 10^-8^ M mixture of sulphated and non-sulphated Myc-LCOs (*n* = 3 independent pools of 10 plants). **(C)** Quantification of *NIN* expression in plants treated (CO4) or not (control) with 10^-8^ M of CO4 (*n* = 3 independent pools of 10 plants). Error bars represent SEM, asterisk indicates a significant difference between the two treatments according to the Mann–Whitney test (*p* < 0.05).

Nodule Inception is known for many years to play a key role during nodulation (Schauser et al., 1999). Analysis of *NIN* expression using the Medicago gene atlas^2^ reveals that *NIN* is induced during this symbiosis and by exogenous Nod factor treatment. Interestingly, the Medicago gene atlas also reveals that *NIN* expression is induced in 3-week-old mycorrhized roots and after Myc-LCOs treatment. We first confirmed by qRT-PCR that the expression of *NIN* was increased (2.5-fold compared to the control) during mycorrhization (**Figure 1A**). In parallel, we also checked whether a mix of Myc-LCOs (sulphated and non-sulphated Myc-LCOs) could induce *NIN* expression. In order to inhibit the Nod signaling pathway, and to avoid crosstalk with the Myc pathway, we grew the plants under conditions of high nitrogen fertilization (10 mM NH_4_NO_3_), and we saw that *NIN* expression was induced by the Myc-LCOs (**Figure 1B**). Moreover, this induction was still present in the *ram1* mutant but not in the *ipd3* mutant and *nsp1* mutant (**Figure 1B**), suggesting that the induction of *NIN* expression by Myc-LCOs is independent of RAM1 but requires IPD3 and/or NSP1–NSP2, which act upstream of NIN in the Nod signaling pathway (Horváth et al., 2011). Interestingly, we found no induction of *NIN* expression by treatment with CO4 (**Figure 1C**), confirming that the symbiotic signaling pathways induced by Myc-LCOs and COs are distinct (Genre et al., 2013).

### Mycorrhization Is Affected in the Medicago *nin* Mutant

**FIGURE 2:**
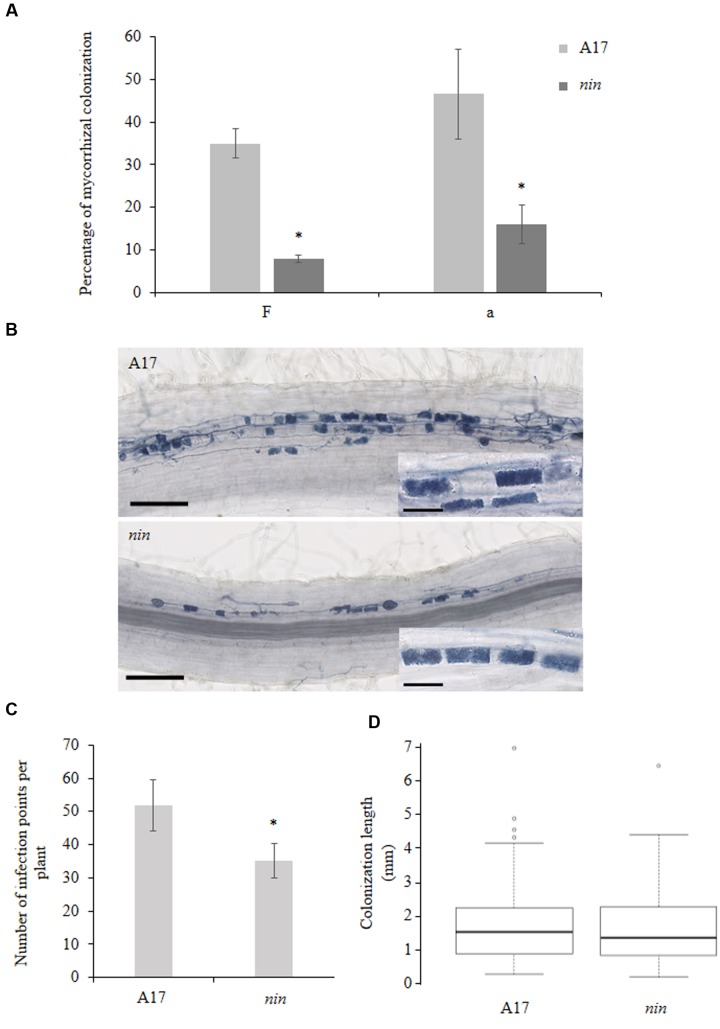
**Mycorrhizal phenotype of the *nin* mutant in *M. truncatula*.** **(A)** Percentage of colonization in the roots of WT A17 and *nin* mutant cultivated for 9 weeks according to the Trouvelot’s method (Trouvelot et al., 1986). ‘F’: frequency of colonization in the root system; ‘a’: arbuscule abundance (in percentage) in the colonized root sections. **(B)** Representative fungal colonization pattern in A17 WT strain and *nin* mutant, 2 weeks post inoculation. **(C)** Quantification of infection points per plant in WT A17 and *nin* mutant, 2 weeks after inoculation with 50 spores per plant. **(D)** Boxplot representing the fungal colonization length from each infection point, in WT A17 and *nin* mutant, 2 weeks after inoculation with 50 spores per plant. Error bars represent SEM. Asterisks indicate a significant difference when compared to control according to the Kruskal–Wallis test (*n* = 8, *p* < 0.05).

Because the use of high spore inoculum could hide some mycorrhization phenotypes (Maillet et al., 2011; Delaux et al., 2013), we used a small inoculum (400 spores per liter of substrate) to investigate the mycorrhizal phenotype of the *Mtnin-1* mutant (Horváth et al., 2011; Vernié et al., 2015). After 9 weeks, mycorrhization of mutant plants was significantly reduced compared to the wild-type (WT; **Figure 2A**). More precisely, both the frequency of colonization (F) as well as the abundance of arbuscules (a) in the colonized root part were reduced of about fourfold compared WT plants (**Figure 2A**). Nevertheless, despite this strong reduction of the colonization, the arbuscule structure appeared unaffected (**Figure 2B**). In addition, we analyzed the *Mtnin-1* mycorrhizal pattern at very early stages of fungal colonization (2 weeks post inoculation). To do so, we analyzed the number of fungal entrances per plant (infection points), and the distance of internal hyphae propagation from each infection point. We detected a lower number of infection points in the *Mtnin-1* mutant compared to WT plants (**Figure 2C**), whereas the internal propagation of hyphae did not shown any obvious defect (**Figure 2D**), suggesting that NIN is required for efficient penetration of the fungus, rather than involved in the control of the fungal colonization of the root.

**FIGURE 3:**
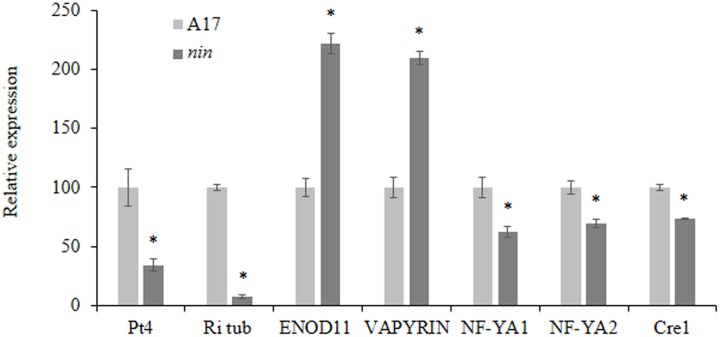
**Expression analysis by RT-qPCR of marker genes in *M. truncatula* WT A17 and *nin* mutant roots cultivated for 9 weeks and inoculated by *R. irregularis*.** Ri tub, *R. irregularis* tubulin, Pt4, Phosphate transporter 4, ENOD11, EARLY NODULIN 11. Error bars represent SEM. Asterisks indicate a significant difference when compared to control according to the Kruskal–Wallis test (*n* = 6, *p* < 0.05).

Nodule Inception have already been described as involved in the regulation of *ENOD11*, a marker gene for early symbiotic responses during nodulation, by competitive inhibition of the transcription factor ERN for induction of the NF-box within the *ENOD11* promoter (Vernié et al., 2015). In parallel, NIN has been shown to activate expression of several genes involved in nodule biogenesis (Laloum et al., 2014; Vernié et al., 2015). To analyze if these activities are still present during mycorrhization, we assessed by qRT-PCR the expression of these genes in the *Mtnin-1* mutant. We also checked the expression of some marker genes such as the *R. irregularis* tubulin and the plant *VAPYRIN* gene, coding for a protein involved in vesicle trafficking during mycorrhization, essential for arbuscule formation and for efficient epidermal penetration of AM fungi (Pumplin et al., 2010). To ensure the good functionality of arbuscules, we assayed the expression of a phosphate transporter *MtPt4*, up-regulated during mycorrhization and necessary for the development of fully active arbuscules (Harrison et al., 2002; Volpe et al., 2016). Confirming the reduced mycorrhizal phenotype, fungal development markers were less expressed in *Mtnin-1* mutant reflecting the general lower mycorrhization rate in these plants (**Figure 3**). Interestingly, *ENOD11* and *VAPYRIN* expression was increased in *Mtnin-1* mutant, suggesting that NIN might also display an inhibitory activity on these promoters in during mycorrhization, whereas expression of genes described as NIN targets, *NF-YA1*, *NF-YA2*, and *CRE1* (Soyano et al., 2013; Vernié et al., 2015) was slightly repressed, suggesting that the regulation of gene expression by NIN is similar between nodulation and mycorrhization.

## Discussion

It is presently known that NIN is of paramount importance as a central regulator of nodulation. First of all, it plays essential roles locally in the root epidermis where it is necessary for the initiation of bacterial infection, and in the root cortex by promoting nodule organogenesis, through induction of cytokinin receptor *CRE1* expression (Schauser et al., 1999; Soyano et al., 2013; Vernié et al., 2015). In this context, it should be interesting to redefine more precisely the firstly described mycorrhizal phenotype of *cre1* mutant, by using smaller inoculum (Laffont et al., 2015). Moreover, NIN acts in a more systemic manner as a negative regulator, inhibiting additional bacterial responses after the initial activation of the CSSP and promoting autoregulation of nodulation by inducing CLE peptides that limits the final number of nodules (Marsh et al., 2007; Soyano et al., 2014; Yoro et al., 2014).

Here, we demonstrate the involvement of NIN in the AM symbiosis showing that its impairment leads to a decrease of the total fungal colonization and the number of infection points. However, although NIN seems to be important for an efficient mycorrhization, its mutation does not lead to a defect in arbuscule morphogenesis (**Figure 2B**). This phenotype is very similar to those observed in mutants of different members of the CSSP, like *IPD3*, *NSP1*, or *NSP2* (Horváth et al., 2011; Maillet et al., 2011; Delaux et al., 2013). However, it would be very interesting to phenotype in more details these mutants, i.e., for their ability of colonization and infection. This would allow to check whether the corresponding genes are involved in the same mycorrhizal process. Interestingly, like in nodulation, we found that NIN was important for *ENOD11* down-regulation during mycorrhization. This highlights a possible role in the regulation of recognition and signaling pathways occurring during primary steps of fungal entrance. Moreover, the *VAPYRIN* gene, which is crucial for fungal development and morphogenesis in the roots, is also up-regulated during mycorrhization in the *nin* mutant compared to the WT. In addition to ENOD11, this indicates a possible role of NIN acting by tempering the expression of some crucial genes leading to a control of the fungal development in the roots.

Interestingly the fact that the *nin* mutant displays a lower number of infection points suggests that NIN could, when induced by Myc-LCOs, functions to allow entry of mycorrhizal fungi into the root. We could speculate that NIN could be involved in the complex control of phytohormones like strigolactones, crucial for fungal stimulation in the rhizosphere and fungal entrance. Indeed, strigolactones biosynthesis is controlled by *NSP1*, a GRAS transcription factor acting upstream of *NIN* (Marsh et al., 2007; Middleton et al., 2007; Liu et al., 2011; Cerri et al., 2012). It then would be interesting to better investigate if *nin* is affected in the strigolactone biosynthesis.

Moreover, NIN has been shown to be involved in the regulation of a NPL crucial for bacterial infection (Xie et al., 2012). Indeed this enzyme seems to be necessary for cell wall modifications allowing bacteria entrance. However, since we could not found any difference in fungal propagation between the *nin* mutant and the control plants (**Figure 2D**), neither than aborted appressoria, we could speculate that the regulation of this enzyme is not involved during mycorrhization.

Altogether, our results point out a complex role of NIN firstly necessary for optimal fungal entry and development, but then acting as a negative regulator in later steps of the colonization by repressing further fungal associated signaling genes.

However, a lot of questions remain to be answered about the potential NIN partners or target genes, and more specifically its role in the autoregulation process during mycorrhization.

## Author Contributions

J-PC designed research, BG, J-MC, and J-PC performed experiments, BG and J-PC wrote the manuscript.

## Conflict of Interest Statement

The authors declare that the research was conducted in the absence of any commercial or financial relationships that could be construed as a potential conflict of interest.

## Footnotes

1 http://www.damolin.fr

2 http://mtgea.noble.org/v3/, probe set: Mtr.28094.1.S1_at

## Supplementary Material

**TABLE S1 | List of primers used.**
