# Supplementary material for: NIN Is Involved in the Regulation of Arbuscular Mycorrhizal Symbiosis
Source: Front Plant Sci. 2016 Nov 16;7:1704. doi: 10.3389/fpls.2016.01704 (PMC5110543; doi:10.3389/fpls.2016.01704)
Supplement: Supplementary file 1 [file Table_1.PDF]

Primer name	Sequence	Reference
MtUbiquitin 5'	GCAGATAGACACGCTGGGA	Lauressergues et al., 2012
MtUbiquitin 3'	AACTCTTGGGCAGGCAATAA	
MtENOD11 5'	AGGTGGAGGCTGGTTACCATAAG	
MtENOD11 3'	GCCTACCACACAAACAACCATCA	
Mtvapyrin 5'	CATCACCACTTGGCACATTC	
Mtvapyrin 3'	GTGAAGGGGTTTGGTGAAGA	
Ritub 5'	TGCCACCATCAAGACCAAA	
Ritub 3'	AAGATCACCACCAGGGACAA	
MtEF1 5’	CTTTGCTTGGTGCTGTTTAGATGG	Gonzalez-Rizzo et al., 2006
MtEF1 3’	ATTCCAAAGGCGGCTGCATA	
MtCre1 5’	CACCACCCTTTGGCTTCTAA	
MtCre1 3’	CACTAAGTAGCGGCCTTTCG	
MtNIN 5'	GTGCTTTCAGAGTGAAGGCAACTT	Moreau et al., 2011
MtNIN 3'	AAATCCCCACATTGCTTGCA	
MtPt4 5'	ACGTTCTTGGTGACGGAAAC	Harrison et al., 2002
MtPt4 3'	GAGCCCTGTCATTTGGTGTT	
MtNF-YA1 5’	AAAATATGGCTATGCAACCTGTTTA	Combier et al., 2006
MtNF-YA1 3’	CAACTGACATCTTACAATCATCTGG	
MtNF-YA2 5’	CGTCGATCACCTTGGATCTT	Laloum et al., 2014
MtNF-YA2 3’	GCCAAAGTAATGAGCCAGGA	

Combier, J.-P., Frugier, F., de Billy, F., Boualem, A., El-Yahyaoui, F., Moreau, S., et al. (2006). MtHAP2-1 is a key transcriptional regulator of symbiotic nodule development regulated by microRNA169 in Medicago truncatula. *Genes Dev.* 20, 3084–8. doi:10.1101/gad.402806.
Gonzalez-Rizzo, S., Crespi, M., and Frugier, F. (2006). The Medicago truncatula CRE1 cytokinin receptor regulates lateral root development and early symbiotic interaction with Sinorhizobium meliloti. *Plant Cell* 18, 2680–2693. doi:10.1105/tpc.106.043778.
Harrison, M. J., Dewbre, G. R., and Liu, J. Y. (2002). A Phosphate Transporter from Medicago truncatula Involved in the Acquisition of Phosphate Released by Arbuscular Mycorrhizal Fungi. *PLANT CELL ONLINE* 14, 2413–2429. doi:10.1105/tpc.004861.
Laloum, T., Baudin, M., Frances, L., Lepage, A., Billault-Penneteau, B., Cerri, M. R., et al. (2014). Two CCAAT-box-binding transcription factors redundantly regulate early steps of the legume-rhizobia endosymbiosis. *Plant J.* 79, 757–768. doi:10.1111/tpj.12587.
Lauressergues, D., Delaux, P.-M., Formey, D., Lelandais-Brière, C., Fort, S., Cottaz, S., et al. (2012). The microRNA miR171h modulates arbuscular mycorrhizal colonization of Medicago truncatula by targeting NSP2. *Plant J.* 72, 512–522. doi:10.1111/j.1365-313X.2012.05099.x.
Moreau, S., Verdenaud, M., Ott, T., Letort, S., de Billy, F., Niebel, A., et al. (2011). Transcription reprogramming during root nodule development in Medicago truncatula. *PLoS One* 6. doi:10.1371/journal.pone.0016463.

Table S1
